# Optimizing transducer choice for the detection of alveolar–interstitial syndrome in dogs and cats: an evidence-based review

**DOI:** 10.3389/fvets.2026.1778331

**Published:** 2026-04-30

**Authors:** Kyle L. Granger, Cody I. Riffe, Søren R. Boysen, Charles T. Talbot

**Affiliations:** 1 Emergency & Critical Care Services, Thrive Pet Healthcare Specialists, Hoffman Estates, IL, United States; 2 Emergency & Critical Care Services, BluePearl Veterinary Partners, Nashville, TN, United States; 3 Department of Veterinary Clinical and Diagnostic Sciences, Faculty of Veterinary Medicine, University of Calgary, Calgary, AB, Canada; 4 Department of Clinical Sciences, College of Veterinary Medicine and Biomedical Sciences, Colorado State University, Fort Collins, CO, United States

**Keywords:** AIS, alveolar–interstitial syndrome, B-lines, cats, dogs, lung ultrasound, pleural abnormalities, point-of-care ultrasound

## Abstract

Alveolar–interstitial syndrome (AIS) is a common ultrasonographic finding in dogs and cats with pulmonary pathology, including cardiogenic pulmonary edema, noncardiogenic pulmonary edema, pneumonia, acute respiratory distress syndrome, and pulmonary contusion. Point-of-care lung ultrasound (LUS) has emerged as a rapid, noninvasive, and repeatable diagnostic tool for identifying AIS in emergency and critical care patients. Despite its increasing clinical adoption, transducer selection remains largely operator-dependent, with limited evidence-based guidance for veterinary applications. This review synthesizes current veterinary and human literature to evaluate how transducer type influences the detection and characterization of AIS in small animals. The acoustic properties, field of view, penetration depth, and resolution trade-offs of linear, curvilinear, and microconvex transducers are discussed in relation to B-line visualization, pleural line assessment, and detection of subpleural consolidations. Comparative performance data, including the results of recent veterinary studies, are integrated to highlight differences in diagnostic sensitivity, specificity, and interobserver reliability. A clinical decision-making framework is proposed, outlining optimal transducer selection by patient size, suspected pathology, and clinical context (triage, monitoring, follow-up). Practical considerations such as transducer availability, equipment ergonomics, and operator training are addressed. Knowledge gaps and future research priorities, including the role of portable devices and AI-assisted image interpretation, are identified. By consolidating the available evidence, this review aims to standardize and optimize transducer selection in veterinary lung ultrasound, improving diagnostic accuracy, accelerating clinical decision-making, and ultimately enhancing patient outcomes in small animal emergency and critical care.

## Introduction

Alveolar–interstitial syndrome (AIS) is a lung ultrasound pattern that arises when normally aerated lung is replaced by fluid, cells, or pathological tissue (1–7). Alteration of the pleural–subpleural interface under these conditions produces B-lines, pleural irregularities, and subpleural consolidations, also referred to as tissue-like patterns (1–7). AIS has been described across a broad spectrum of conditions, including cardiogenic and noncardiogenic pulmonary edema, acute respiratory distress syndrome, pneumonia, pulmonary contusions, atelectasis, interstitial fibrosis, neoplastic infiltration, and chronic interstitial lung diseases (1–7). Human literature further reports B-lines in scleroderma-associated interstitial lung disease, cystic fibrosis pulmonary exacerbations, and other chronic parenchymal disorders, reinforcing that AIS is not specific to fluid overload (8–12). Overlooking these dry causes can lead to premature or inappropriate diuretic administration in both human and veterinary settings (13–18).

Human clinical studies demonstrate that lung ultrasound detects interstitial abnormalities earlier and with greater sensitivity than radiography and correlates closely with computed tomography (CT) (1, 4, 5, 7, 15). Its portability, repeatability, and suitability for rapid reassessment have supported its increasing use in small animal emergency and critical care. Veterinary studies in dogs and cats demonstrate reproducible associations between B-lines, pleural abnormalities, subpleural tissue-like patterns, and pulmonary disease (16, 19–22).

Transducer selection (colloquially referred to as “probe” selection) remains variable across veterinary practice. Microconvex or curvilinear transducers are commonly used, whereas linear transducers dominate pleural assessment in human lung ultrasound and phased-array transducers are typically chosen for integrated cardiopulmonary imaging (23–26). Although transducer frequency, footprint, and beam geometry influence pleural-line detail, near-field resolution, and intercostal access, these characteristics do not alter the ability to generate AIS-defining artifacts (7, 19, 27).

Comparative veterinary studies show that linear, microconvex, curvilinear, and phased-array transducers all produce diagnostically valid B-lines and subpleural tissue-like patterns despite expected differences in artifact appearance (4, 14). Cross-transducer evaluations confirm consistent AIS detection across these transducer types (14, 28). These findings parallel human consensus statements and clinical studies, which classify AIS identification as transducer-agnostic (3, 4, 7). Selecting a transducer that aligns with patient conformation, thoracic depth, and the diagnostic aim of the examination can improve image quality and reduce variability between operators and clinical environments.

The purpose of this review is to synthesize human and veterinary evidence regarding the performance of linear, microconvex, curvilinear, and phased-array transducers for AIS detection in dogs and cats. The review outlines the acoustic physics underlying vertical artifact formation, describes how transducer characteristics influence pleural and subpleural visualization, and presents a structured decision pathway for transducer selection in veterinary lung ultrasound (VLUS). Since much of the foundational knowledge regarding lung ultrasound originates from human critical care medicine and respiratory imaging research, this review integrates those data with emerging veterinary studies in dogs and cats, while noting areas in which current interpretation relies primarily on extrapolation from human literature.

## Literature identification

This narrative review synthesizes relevant human and veterinary literature addressing lung ultrasound physics, artifact generation, and the detection of alveolar–interstitial syndrome. Searches were performed in PubMed/MEDLINE, CAB Abstracts, Scopus, Web of Science, and Google Scholar for studies published between 2000 and 2025 using combinations of the terms lung ultrasound, B-lines, alveolar–interstitial syndrome, pneumonia, interstitial lung disease, dogs, cats, transducer, and point-of-care ultrasound. Studies examining artifact physics, diagnostic performance of lung ultrasound in pulmonary disease, and comparisons of transducer types or imaging parameters were prioritized. Both human and veterinary studies were included, with emphasis on research involving dogs and cats where available.

## Understanding AIS

Alveolar–interstitial syndrome (AIS) encompasses a group of lung ultrasound findings that develop when normally aerated lung is partially or completely replaced by fluid, cells, or pathological tissue. These alterations modify how ultrasound energy interacts with the pleural–subpleural interface. In healthy dogs and cats, the pleural line is smooth, thin, and highly reflective, producing horizontal reverberation artifacts (A-lines) that indicate normal aeration (13, 29). The pronounced acoustic mismatch between soft tissue and air prevents ultrasound penetration beyond the lung surface. As interstitial or alveolar density increases, this interface changes and generates vertical artifacts known as B-lines (13, 17). B-lines arise from the pleural line, move with lung sliding, extend to the far field without fading, and erase A-lines, which makes them a reproducible indicator of reduced aeration (30).

Both human and veterinary literature emphasize that AIS is not restricted to “wet lung” conditions. Although extravascular lung water from cardiogenic pulmonary edema, acute respiratory distress syndrome, and noncardiogenic edema remains a common cause, numerous dry interstitial processes also produce B-lines and pleural abnormalities. These include atelectasis, fibrotic interstitial remodeling, immune-mediated or inflammatory disease, and certain neoplastic infiltrates (13, 16, 27, 31–36). Recognizing this broader differential list aligns with human consensus recommendations that encourage inclusive interpretation of interstitial artifacts and reduces diagnostic oversimplification (27, 33–35). In both species, attributing AIS solely to fluid overload risks premature or inappropriate diuretic administration.

The physics underlying AIS reflect the creation of “acoustic traps” within partially aerated lung tissue (30, 35). As air content decreases and fluid or tissue replaces alveolar gas, ultrasound energy begins to enter the subpleural parenchyma, where it reverberates between structures of differing impedance (30). This mechanism produces the stable vertical artifacts recognized as B-lines. Human imaging studies show that such artifacts correlate with CT findings including ground-glass opacities, thickened interlobular septa, and early alveolar filling. Veterinary patients with pulmonary edema, pneumonia, interstitial disease, or mixed pathology demonstrate comparable sonographic-radiologic patterns (13, 32, 36–41).

Pleural line abnormalities form a second core component of AIS evaluation. Loss of subpleural aeration from inflammation, fibrosis, edema, or atelectasis can produce an irregular, thickened, or fragmented pleural interface (42). These changes are increasingly recognized as important markers in both acute and chronic lung disease. Small hypoechoic subpleural consolidations, also referred to as tissue-like patterns, represent localized areas of nonaerated lung immediately beneath the pleural surface (13, 39). They occur in pneumonia, pulmonary contusions, aspiration injury, viral processes, and focal noncardiogenic edema (37–41). When combined with pleural abnormalities and B-lines, subpleural consolidations improve the specificity of lung ultrasound for differentiating AIS etiologies.

Collectively, B-lines, pleural irregularities, and subpleural consolidations define the sonographic signature of AIS. The mechanisms responsible for these findings are conserved across species because canine and feline lungs share air–tissue acoustic properties with human lungs (5, 13). This cross-species consistency supports the adaptation of human interpretive frameworks to veterinary practice. Lung ultrasound enables real-time assessment of pulmonary aeration, allowing clinicians to distinguish diffuse from focal disease, monitor dynamic cardiogenic or inflammatory change, and guide targeted diagnostic and therapeutic decision-making in emergency and critical care.

## Transducer types and characteristics

Ultrasound transducers vary in frequency, penetration, resolution, footprint geometry, and field of view, and these characteristics influence artifact appearance and pleural visualization during lung ultrasound. In small animal medicine, the transducers most commonly used for thoracic imaging are microconvex, linear, and phased-array units (3). Microconvex transducers remain the predominant choice for general lung ultrasound in dogs and cats, while linear transducers are used frequently for high-detail pleural assessment. Phased-array transducers are used less commonly for primary lung imaging but are selected when cardiac and pulmonary examinations must be performed concurrently. This order is consistent with available veterinary literature, including cross-transducer comparisons in dogs and cats and recent experimental analyses of vertical artifact morphology (4, 14, 19).

The primary consideration in transducer selection is the relationship between frequency and penetration. Higher frequencies improve near-field structural detail but limit depth, while lower frequencies increase penetration at the cost of fine resolution (43). Because lung ultrasound requires visualization of both the pleural line and deep acoustic artifacts, no universal recommendation exists for an ideal transducer in small animals. Human lung ultrasound guidelines often recommend using both a convex or microconvex transducer and a high-frequency linear transducer when available to evaluate superficial and deeper thoracic structures (14, 28). Veterinary studies similarly demonstrate consistent interpretation of B-lines, pleural abnormalities, and subpleural tissue-like patterns across microconvex, linear, and phased-array transducers when used by experienced operators (4, 14, 19).

**Figure 1:**
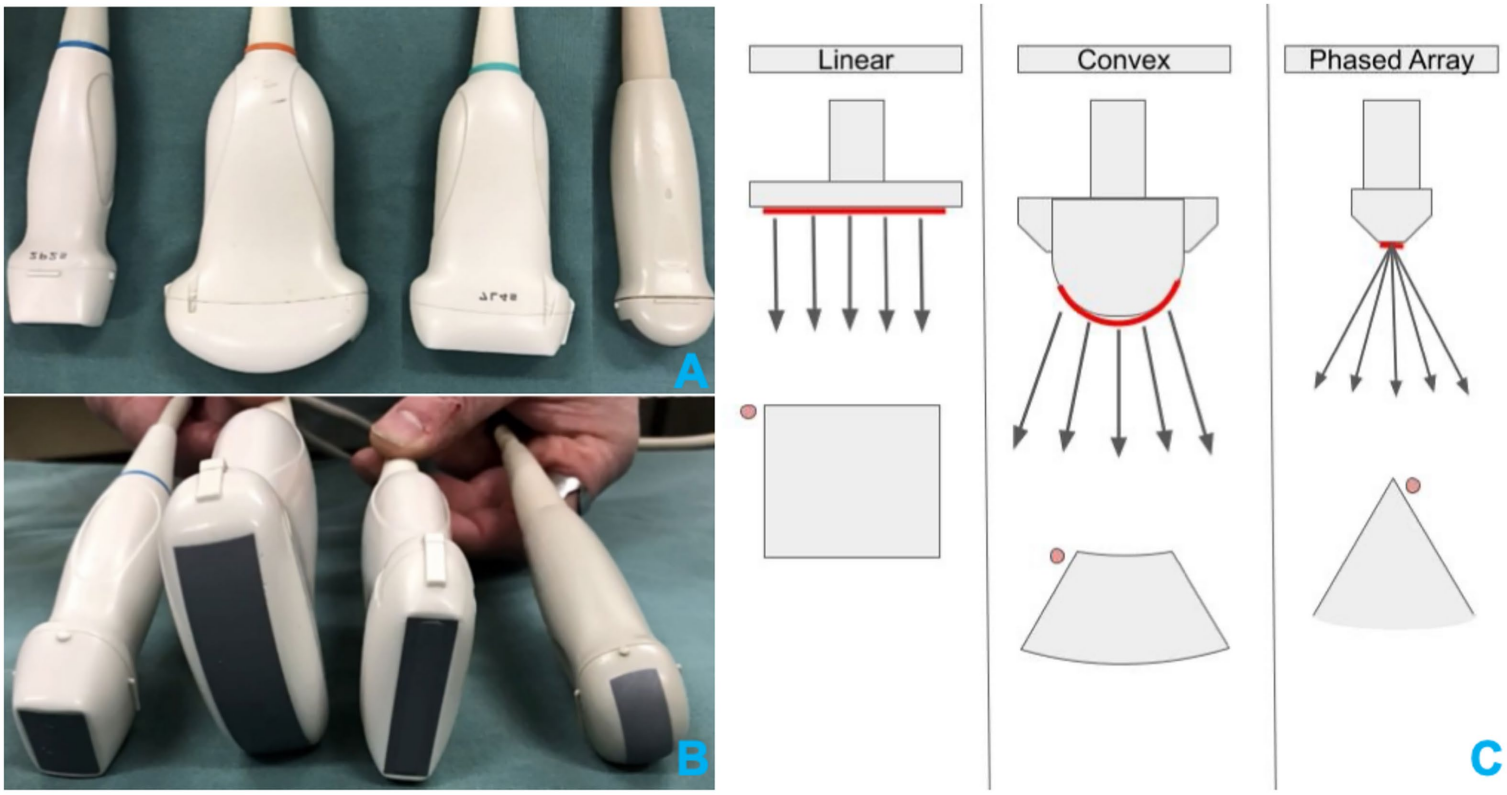
Representative ultrasound transducers and corresponding beam geometries used in small-animal lung ultrasound. **(A,B)** Show the external appearance and footprint surfaces of linear, curvilinear, microconvex, and phased-array transducers. These images illustrate true transducer dimensions and clarify that phased-array footprints are not always markedly smaller than microconvex transducers, despite traditional schematic depictions. **(C)** Presents simplified beam-geometry diagrams for each transducer type, highlighting differences in footprint shape, field of view, and near-field characteristics. Together, the panels link real transducer morphology with conceptual beam behavior to assist readers in understanding how transducer design influences pleural access, intercostal maneuverability, and artifact appearance during lung ultrasound transducer dimensions vary across manufacturers and models; the Transducers illustrated represent common configurations but may not be drawn to exact scale.

Most transducers used for small animal thoracic imaging function between approximately 3 and 12 MHz. Frequencies above 12 MHz exist commercially, particularly among ultra-high-frequency linear transducers, but are less common in routine clinical practice because they limit penetration and field of view (24, 44–46). Common transducer categories, frequency ranges, and clinical characteristics are summarized in Table 1. Differences in beam geometry, footprint, and external appearances are illustrated in Figure 1.

**Table 1:** Technical and clinical performance profiles of common ultrasound transducers used for alveolar–interstitial syndrome assessment (25, 32, 76).

Transducer type	Typical frequency range (MHz)	Field of view	Advantages for AIS detection	Limitations	Representative evidence
Linear	7–15	Rectangular field with large near-field zone	Highest pleural-line resolution; optimal for pleural irregularities, early subpleural lesions, and fine vertical artifacts	Limited penetration in large dogs; reduced artifact brightness at depth	High-frequency linear transducers improve pleural assessment; AIS detection remains transducer-agnostic
Microconvex	4–8	Small, curved footprint offering wide intercostal access	Balanced resolution and penetration; strong vertical artifact generation; versatile for whole-lung scanning; preferred for most small animals	Slightly reduced near-field detail compared with linear; changes in frequency may alter artifact thickness	Widely used for global lung scanning; supported in veterinary and human protocols
Curvilinear	2–6	Larger curved footprint with deep penetration	Strong vertical artifact formation in deeper regions; useful for medium to large dogs and overweight patients	Reduced pleural-line sharpness; larger footprint may limit intercostal access	Lower-frequency transducers create thicker vertical artifacts but maintain diagnostic validity
Phased-array	1–5	Narrow sector footprint optimized for cardiac and thoracic access	Excellent penetration; efficient rib-space navigation; useful for integrated cardiopulmonary POCUS and dyspneic patients	Reduced near-field clarity; less sensitive for small pleural defects or early irregularities	Reliable for B-line and consolidation detection in emergency and critical care settings

The following subsections summarize the characteristics, strengths, and limitations of the main transducer types used for VLUS.

### Linear transducers

Linear transducers operate at high frequencies, typically 7 to 18 MHz, and produce a rectangular field of view beneath a long, straight footprint (47, 48). Their parallel beam geometry generates a uniform near-field image and provides excellent pleural-line resolution (48). This makes the linear transducer the most sensitive option for detecting subtle pleural irregularities, early subpleural lesions, and fine vertical artifacts. In dogs and cats, B-lines visualized with linear transducers often appear thin at the pleural origin with limited distal widening, a finding that aligns with controlled comparisons of artifact morphology across transducer types (14).

Linear transducers are used for pleural assessment, musculoskeletal imaging, vascular access, and evaluation of superficial abdominal organs (49). The elongated footprint can be helpful for maintaining transducer stability between ribs. Although some operators report discomfort in thin or painful patients when perpendicular rib pressure is required, this is primarily an operator-dependent issue rather than an intrinsic limitation of the transducer (50). Limited penetration restricts use of linear transducers for assessing deeper pulmonary or abdominal structures in large dogs.

### Microconvex transducers

Microconvex transducers have a small, curved footprint that produces a sector-shaped field of view. They typically operate between 3 and 12 MHz and offer a balance of near-field detail and depth penetration appropriate for most canine and feline thoracic examinations (47). B-lines produced by microconvex transducers widen distally in a fan pattern because of the curvature of the beam (47). This configuration allows simultaneous assessment of the pleural line, lung parenchyma, and deeper interfaces.

Microconvex transducers are widely used in small animal point-of-care ultrasound because they accommodate diverse thoracic conformation, maneuver easily between ribs, and can be used for abdominal and targeted cardiac imaging (14). Veterinary studies report high agreement between microconvex and linear transducers when evaluating B-lines and pleural abnormalities in dogs and cats (4, 14, 19). Recent veterinary studies comparing microconvex, linear, and phased-array transducers describe frequency- and geometry-dependent differences in B-line appearance, although diagnostic agreement remains high (1, 3, 14). Because of their versatility and ergonomic footprint, microconvex transducers are frequently considered the default choice for VLUS.

### Macroconvex transducers (large animal use and fringe small animal use)

Macroconvex transducers have a larger curved footprint and lower frequency range, typically 1 to 4 MHz (47). They are standard in large animal practice because of their ability to penetrate deeply through the adult bovine or equine thorax. These transducers are not commonly used in VLUS and have not been formally evaluated in canine or feline AIS studies. Their primary relevance to small animal clinicians is understanding the distinction from microconvex transducers, which offer similar geometry but with dimensions more appropriate for dogs and cats.

### Phased-array transducers

Phased-array transducers use electronic beam steering to generate a narrow sector-shaped field of view and typically operate between 1 and 13 MHz (47). The small square or triangular footprint provides excellent access between ribs, which is advantageous in tachypneic or recumbent patients. High temporal resolution makes phased-array transducers the preferred option for cardiac imaging.

The narrow near-field sector can reduce pleural-line clarity compared with linear or microconvex transducers. However, phased-array transducers consistently generate diagnostically valid B-lines and subpleural tissue-like patterns in both experimental work and clinical veterinary studies (4, 14). They are commonly used in emergency and intensive care settings where rapid transitions between cardiac and pulmonary windows are required.

### System categories: handheld, portable, and console platforms

Veterinary LUS is performed across a range of ultrasound system types, including handheld devices, portable laptop-style systems, and full cart-based consoles. These systems differ in image quality, processing capability, transducer availability, and ergonomics. Some handheld devices use semiconductor-based chip transducers that allow a single transducer to emulate multiple geometries, while others rely on traditional piezoelectric elements with separate linear and convex transducers.

Handheld units offer practical advantages such as mobility and affordability, although they may be limited by smaller image displays, shorter battery life, overheating in some non-chip systems, and reduced spatial resolution compared with larger platforms. A recent veterinary abstract comparing three handheld devices demonstrated meaningful differences in image quality and artifact clarity across brands (51). Portable laptop-style systems provide improved processing speed and larger screens, while console platforms offer the highest and most consistent image quality for thoracic and cardiac imaging.

Performance differences between machine categories can exceed differences between individual transducers. Recognizing these system-level limitations helps clinicians maintain realistic expectations during lung ultrasound acquisition and interpretation.

## Evidence from human medicine

Human LUS research provides the most extensive evidence regarding how transducer selection influences the detection and interpretation of AIS. Foundational studies established B-lines as hyperechoic vertical artifacts arising from the pleural line, extending to the far field without fading, erasing A-lines, and correlating with reduced aeration on CT, including thickened interlobular septa, ground-glass opacity, and early alveolar flooding (25, 33, 52, 53). These early findings positioned LUS as a sensitive tool for identifying cardiogenic edema, ARDS, pneumonia, and diffuse interstitial disease well before widespread veterinary adoption (25, 33, 52–54).

### Clinical applications of lung ultrasound

Across acute respiratory failure, emergency presentations, trauma, intensive care monitoring, and cardiology, LUS consistently outperforms chest radiography for early detection of interstitial patterns (54–57). B-lines correlate with extravascular lung water and often appear earlier than radiographic abnormalities, facilitating faster clinical decision-making (54, 55, 57). Serial LUS is now routine in human critical care for monitoring therapeutic response, fluid status, ventilator induced conditions, and disease progression (25, 33, 53). Collectively, these applications have established lung ultrasound as a central bedside imaging modality in human emergency and critical care practice.

### Transducer independence in AIS detection

A central conclusion from human research is that AIS detection is effectively transducer-agnostic. Linear, curvilinear, microconvex, and phased-array transducers all generate diagnostic B-lines, and major consensus statements, including those from the International Liaison Committee on Lung Ultrasound (ILC-LUS), affirm that no single transducer is inherently superior for identifying AIS (16). Although transducers differ in near-field resolution, penetration, and ergonomics, all reliably visualize vertical artifacts when imaging partially aerated lung (16, 33, 45). This principle supports translational application to veterinary medicine, where transducer availability varies widely.

Transducer-specific advantages refine image detail rather than determine diagnostic capability. High-frequency linear transducers optimize pleural-line assessment, including pleural fragmentation and small subpleural consolidations, whereas curvilinear and phased-array transducers improve penetration and global evaluation of diffuse AIS patterns (16, 45). Microconvex transducers balance these properties and are widely used in human and veterinary POCUS for their ergonomic versatility (16, 45).

Experimental human studies explain the consistency of AIS detection across transducers by confirming that B-lines originate from “acoustic traps” in partially aerated lung tissue rather than from transducer geometry (9, 53). Although artifact thickness or brightness varies with frequency and beam characteristics, the presence of B-lines remains a stable indicator of interstitial pathology across devices (9, 53). Cardiologic studies further demonstrate transducer independence, showing that B-line counts correlate with extravascular lung water and provide prognostic information independent of radiography, natriuretic peptides, or echocardiographic findings (56). These findings demonstrate that transducer characteristics influence artifact appearance and image detail but do not determine the fundamental ability to detect AIS.

### Reproducibility and protocol development

Interobserver reliability is another strength of the human literature. Simplified scanning protocols yield high agreement between novice and expert operators in detecting B-lines and pleural abnormalities (58–60). This reproducibility has supported recommendations that LUS be used as a first-line imaging modality for acute dyspnea and respiratory failure in emergency and intensive care settings (16, 53, 54, 59).

Human evidence also emphasizes the importance of appropriate machine settings. Excessive harmonic imaging, artifact suppression, or suboptimal dynamic range can reduce B-line visibility, especially on portable or handheld platforms (33). These considerations are directly relevant to veterinary clinicians working with diverse ultrasound systems because settings may influence artifact appearance as much as transducer type.

Overall, human LUS data show that while transducer characteristics affect image detail and ergonomics, the fundamental ability to detect AIS is consistent across all major transducer types. These findings form the basis for veterinary adaptation of transducer-selection principles and support the conclusion that optimizing transducer choice enhances image quality and confidence but does not alter the core diagnostic capacity of LUS for AIS.

## Evidence from veterinary medicine

Veterinary LUS research has expanded substantially over the past decade, providing increasing clarity regarding how transducer selection influences detection of AIS in dogs and cats. Early studies demonstrated the diagnostic utility of VLUS for pulmonary edema, pneumonia, aspiration injury, contusions, and pleural space disorders, but they offered limited insight into transducer performance (5, 7, 13, 19). Transducer choice in these investigations often reflected equipment availability rather than standardized methodology, resulting in the use of sector/phased-array cardiac transducers, convex abdominal transducers, and occasional linear transducers (5, 13). This pragmatic approach confirmed that LUS reliably identifies B-lines and subpleural pathology across transducer types, but it did not establish whether specific transducers confer diagnostic advantages.

### Emergence of transducer-specific comparative studies in veterinary medicine

Recent veterinary research has begun to address this knowledge gap, evaluating how microconvex, linear, and phased-array transducers differ in their ability to depict vertical artifacts, pleural-line features, and subpleural lesions.

#### Microconvex vs. phased-array in cardiogenic pulmonary edema

Ward et al. (3) prospectively compared microconvex and phased-array transducers in dogs with acute left-sided congestive heart failure. Both transducers reliably identified B-lines and demonstrated excellent interobserver agreement for quantitative B-line counts (*κ* > 0.8). Image quality, however, was consistently rated higher with the microconvex transducer, which offered clearer anatomic landmarks and more intuitive orientation. These findings indicate that while either transducer can diagnose cardiogenic pulmonary edema, the microconvex transducer may enhance interpretability, especially for teaching, orientation, and serial monitoring.

#### Agreement across microconvex, linear, and phased-array transducers

In one of the largest comparative veterinary LUS studies to date, Łobaczewski et al. (4) evaluated 200 dogs and cats with respiratory distress using microconvex, linear, and phased-array transducers. Agreement between the microconvex and linear transducers was exceptionally high (weighted *κ* > 0.90), supporting their interchangeable use in clinical settings. Pairings involving the phased-array transducer showed only moderate to high agreement (κ ~ 0.60–0.90), with occasional undercounting of B-lines in specific regions. These discrepancies likely reflect the phased-array’s lower near-field resolution and narrower beam footprint. The same study introduced a lung ultrasound scoring system correlating B-line burden with specific etiologies of dyspnea. Quantitative lung ultrasound scoring systems that incorporate regional B-line burden have been increasingly investigated as tools for standardizing interpretation and monitoring disease severity in veterinary patients. Regionally based examinations such as the veterinary bedside lung ultrasound examination (Vet BLUE) divide the thorax into predefined scanning sites and allow clinicians to document the presence and number of B-lines across multiple lung regions (35, 61). More recently, lung ultrasound scoring systems have been proposed in dogs and cats to quantify pulmonary aeration loss and monitor therapeutic response (7, 41). In these approaches, individual thoracic regions are assigned scores based on B-line number, coalescence, or the presence of consolidations, allowing calculation of a cumulative lung ultrasound score that correlates with disease severity and clinical progression (7, 41). Similar quantitative frameworks are widely used in human critical care medicine for monitoring pulmonary edema and acute respiratory distress syndrome and may provide comparable benefits for standardized assessment and longitudinal monitoring in small animal patients. Although diagnostic differentiation is beyond the scope of transducer selection, the scoring system underscores the clinical importance of transducers that accurately capture subtle vertical artifacts.

#### Vertical artifact characteristics across transducers

The recent study by Gajewski et al. (28) provides the most detailed veterinary evaluation of vertical artifact characteristics across microconvex, phased-array, and linear transducers. The authors demonstrated that: (1) All three transducers reliably produced diagnostic hyperechoic vertical artifacts. (2) Artifact morphology differed in predictable ways: linear transducers generated thin, sharply defined artifacts; microconvex transducers produced balanced artifact profiles; and phased-array transducers yielded slightly thicker artifacts attributable to lower frequency and beam convergence. (3) Differences in artifact appearance did not impact diagnostic interpretation of AIS.

This study directly parallels human data showing that while artifact morphology varies with frequency and beam geometry, the presence of B-lines remains consistent across transducers.

#### Pleural line and subpleural pathology across transducers

Granger et al. (1) examined pleural-line and subpleural artifact interpretation using a high-frequency linear transducer versus a curvilinear transducer in dogs. The linear transducer significantly improved interobserver agreement for pleural-line homogeneity (*κ* = 0.89–1.0), whereas agreement with the curvilinear transducer was minimal to fair. High-frequency imaging also enhanced detection of fine subpleural irregularities that were less apparent with the curvilinear transducer. These findings suggest that when the diagnostic question centers on pleural-line pathology or early subpleural lesions, a linear transducer offers superior interpretive consistency.

### AIS detection across disease categories

Beyond comparative studies, veterinary LUS research demonstrates consistent utility across a range of pulmonary diseases:

- Cardiogenic pulmonary edema: diffuse B-lines and dependent consolidations are reliably detected across transducer types (3, 4, 6, 7).
- Pneumonia and aspiration injury: focal or multifocal consolidations correlate well with radiographic and clinical diagnosis (19).
- Pulmonary contusions: LUS identifies early focal B-lines and tissue-like patterns and can exceed radiography in sensitivity (22).
- Noncardiogenic pulmonary edema/ARDS: LUS patterns, including diffuse pleural irregularities and vertical artifacts, are diagnostically informative, though transducer-specific data remain limited (62).
- Chronic cough and inflammatory airway disease: consistent LUS features differentiate affected animals from clinically normal patients (39).

Together, these data indicate that AIS signatures, including B-lines, pleural irregularities, and subpleural consolidations, are consistently identified across transducer types and underscore the importance of standardized terminology and transducer classification to reduce interpretive variability.

### Influence of frequency, footprint, and patient conformation

Transducer selection must account for body size, thoracic conformation, and body condition score (4):

- Microconvex transducers suit most cats and small-to-medium dogs due to excellent rib-space access and balanced penetration.
- Linear transducers provide superior pleural-line resolution and are advantageous when assessing subtle pleural or subpleural changes.
- Curvilinear transducers offer improved penetration for large and/or overweight dogs.
- Linear transducers may be limited in deep-chested or emaciated animals due to contact challenges or inadequate depth.
- Subxiphoid assessment is facilitated by microconvex and phased-array transducers.

### Ultrasound system factors

Artifact appearance is influenced not only by transducer selection but also by ultrasound system type and preset configuration (3, 63, 64).

- Handheld devices may default to harmonic imaging or artifact suppression that reduces B-line visibility.
- Portable laptop-style units vary in beamforming and image processing.
- Larger console systems generally produce more consistent artifact morphology.

Preliminary veterinary abstracts indicate that image quality can vary across handheld devices even with identical transducer types (51). These findings parallel human data and underscore the need for standardized LUS presets in veterinary practice.

### Interobserver reproducibility

Although veterinary interobserver studies are limited, available evidence indicates encouraging reproducibility:

- Standardized scoring systems show good agreement among operators (7).
- Protocol-based approaches demonstrate consistent identification of B-lines and subpleural lesions (61, 65).
- Abdominal POCUS studies show excellent interobserver agreement for peritoneal effusion scoring between observers with different training levels during both standing and lateral examinations (66).
- Comparative canine lung ultrasound work shows consistent pleural-line and subpleural characterization across high-frequency linear and curvilinear transducers, with strong inter-rater agreement for AIS-relevant findings (1).

### Summary of veterinary findings

Current veterinary evidence supports the following conclusions:

1. AIS detection in dogs and cats is effectively transducer-agnostic.
2. Transducer characteristics influence image detail but not fundamental diagnostic capability.
3. Microconvex transducers offer the best versatility for general LUS in small animals.
4. Linear transducers enhance pleural-line and subpleural assessment and should be incorporated when high-resolution detail is needed.
5. Curvilinear and phased-array transducers reliably detect AIS but may provide less near-field detail.
6. Patient conformation and ultrasound system settings can influence artifact appearance as much as transducer choice.
7. Emerging comparative studies justify development of standardized veterinary LUS terminology and transducer-selection guidelines.
8. These data inform the transducer-specific clinical decision framework presented in Section 6.

### Limitations of current veterinary evidence

Although VLUS research has expanded substantially in recent years, several methodological limitations should be considered when interpreting the available data. Many studies involve relatively small cohorts from single referral institutions, which may limit generalizability across broader clinical populations. Case distributions are often weighted toward moderately to severely affected patients presenting to emergency or intensive care settings, introducing potential spectrum bias. Additional variability arises from differences in ultrasound platforms, transducer frequency ranges, presets, and acquisition protocols, including the number and location of thoracic scanning zones. Operator-related factors may also influence image acquisition and interpretation, as formal training pathways and standardized scoring systems are still evolving in veterinary point-of-care ultrasound. Finally, important evidence gaps remain, including limited feline-specific data and relatively few studies evaluating lung ultrasound performance in dogs and cats with conditions such as acute respiratory distress syndrome, chronic interstitial lung disease, and diffuse fibrotic pulmonary processes.

## Clinical decision framework

**Figure 2:**
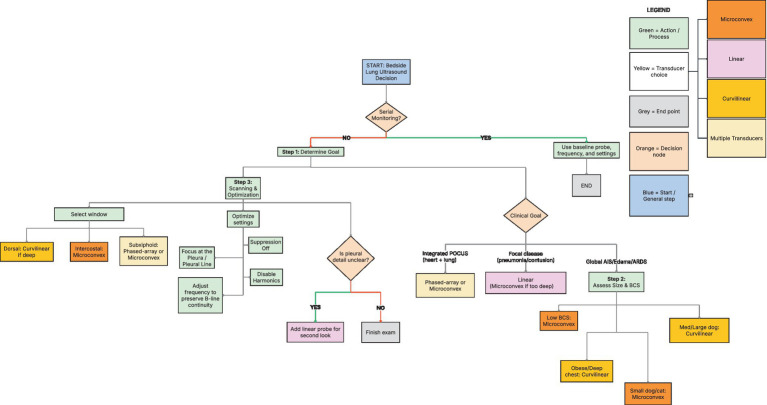
Decision framework for transducer selection and scanning strategy in small-animal lung ultrasound. This flowchart outlines a stepwise approach to transducer choice and image optimization based on clinical objectives, patient morphology, anatomic window requirements, and monitoring needs. The framework emphasizes aligning transducer characteristics with diagnostic goals while maintaining consistency during serial examinations. Key decision nodes incorporate patient-related factors (e.g., body size, thoracic conformation), clinical objectives (e.g., rapid screening, detailed pleural assessment, or monitoring), and equipment considerations (e.g., available transducers and ultrasound platform).

Evidence from human and veterinary studies supports a structured and clinically applicable approach to transducer selection and image interpretation in small animal LUS. Since AIS detection is fundamentally transducer-agnostic and all major transducer types generate diagnostically valid B-lines and pleural abnormalities (5), the framework below prioritizes alignment between transducer characteristics, patient anatomy, diagnostic objectives, ergonomics, and machine capabilities rather than identifying a single preferred transducer. This approach parallels human consensus statements and emerging veterinary data, including recent comparative evaluations of vertical artifact characteristics (5, 13, 37). As illustrated in Figure 2, transducer selection can be guided by clinical objectives, patient morphology, anatomic scanning windows, and equipment availability. Standardized regional scanning approaches further improve reproducibility during lung ultrasound examinations. Protocols such as the modified Armenian and other regionally based scanning schemes divide the thorax into predefined examination sites corresponding to major lung regions, facilitating systematic evaluation and improving interobserver agreement in both dogs and cats (46, 67). Therefore, incorporating consistent scanning locations alongside appropriate transducer selection should enhance comparability between examinations and supports more reliable clinical interpretation.

Moreover, patient stability and positioning must also be considered when performing lung ultrasound in dyspneic or critically ill animals. Whenever possible, examinations should be performed with minimal patient manipulation, often in sternal or standing positions that preserve respiratory mechanics and reduce stress. Regionally based scanning protocols adapted for unstable patients allow clinicians to obtain diagnostically meaningful information while limiting examination time and patient movement. In fact, in a prospective clinical study of dogs and cats with dyspnea, diagnostically consistent B-line detection was achieved across transducers operating at different frequency ranges, including linear (6–14 MHz), microconvex (~5–8 MHz), and phased-array (2–5 MHz) transducers, supporting the use of multiple frequency bands for AIS detection when appropriately matched to patient size and depth requirements (4).

### Step 1: determine the clinical objective

Transducer selection begins with establishing the primary diagnostic purpose of the scan. Objectives generally fall into four categories (4):

#### Global AIS or diffuse respiratory assessment

- Evaluate cardiogenic pulmonary edema, ARDS, noncardiogenic edema, or diffuse interstitial lung disease.
- Lower to mid-range frequencies (approximately 2–8 MHz) were sufficient to generate and quantify B-lines across thoracic regions in dogs and cats, including cardiogenic pulmonary edema and pneumonia, without loss of diagnostic agreement between transducers.
- Recommended transducers: microconvex or curvilinear for depth and field of view.

#### Focal or multifocal parenchymal disease

- Detect pneumonia, aspiration injury, contusions, or focal atelectasis.
- Higher frequencies (approximately 6–14 MHz) permitted detailed pleural-line and subpleural lesion assessment while maintaining high agreement in B-line detection compared with lower-frequency transducers.
- Recommended transducers: linear or microconvex for enhanced pleural detail.

#### Integrated cardiopulmonary POCUS

- Evaluate heart, pleural space, and caudal lung regions, particularly in emergency or ICU settings.
- Recommended transducers: phased-array or microconvex for rapid transitions between cardiac and pulmonary windows.

#### Serial monitoring

- Track B-line burden, consolidation size, ventilator response, or therapeutic effects.
- Recommended approach: maintain the same transducer used at baseline to preserve comparability.

This goal-directed method mirrors human POCUS frameworks and reduces the risk of inferring transducer superiority from context-specific findings.

#### Step 2: assess patient size, thoracic conformation, and body condition score

Patient phenotype strongly influences artifact expression and depth requirements (4, 18, 48, 68).

#### Small dogs and cats

- Microconvex provides optimal intercostal access and adequate depth.
- Linear enhances pleural detail when depth permits.

#### Medium to large dogs

- Curvilinear or mid-microconvex provides adequate penetration.
- Linear can be used in cranial or superficial regions when thoracic depth is favorable.
- Phased-array is suitable when cardiac integration is needed.

#### Very low BCS

- Linear may intermittently lose contact between ribs, creating rib-shadowing artifacts.
- Microconvex maintains better conformity to the thoracic wall.

#### High BCS or obesity

- High-frequency linear transducers may not penetrate adequately.
- Curvilinear or microconvex is preferred for global examination.

#### Deep-chested breeds

- Curvilinear or microconvex provides appropriate depth for AIS assessment.
- Linear may be limited to cranioventral spaces.

This patient-tailored approach aligns transducer function with expected thoracic depth and scanning feasibility. In current veterinary literature, imaging depth was adjusted between 3 and 6 cm depending on patient size, indicating that frequency selection must allow sufficient penetration to this depth to preserve B-line continuity, particularly in larger dogs.

### Step 3: match transducer type to anatomic windows

Different acoustic windows favor different transducer geometries. Thus, anatomical window considerations support maintaining access to at least two transducer types when possible.

#### Intercostal windows

- Microconvex: excellent rib spacing access and versatility.
- Linear: superior pleural detail; restricted by narrow rib spacing or depth.
- Phased-array: good access; reduced near-field pleural resolution.

#### Subxiphoid windows

- Microconvex and phased-array: ergonomic placement under the costal arch and adequate depth for caudodorsal lung surfaces.
- Linear: limited by footprint geometry and penetration in medium and large dogs.

#### Dorsal lung fields

- Thoracic depth often exceeds linear capacity.
- Curvilinear or microconvex is recommended.

If there’s evidence of concurrent pleural effusion, acoustic windows and lung surface visualization may be altered. Pleural fluid can improve visualization of dependent lung surfaces while also affecting the appearance of pleural-line artifacts and subpleural consolidations (46). In such cases, transducers with improved penetration and maneuverability, such as microconvex or phased-array transducer, may facilitate evaluation of deeper lung regions and adjacent pleural structures.

### Step 4: integrate equipment type and machine settings

Machine-related factors can substantially influence artifact visibility, sometimes more than transducer choice.

#### Equipment type

- Handheld units may apply default noise reduction or harmonics that suppress B-lines.
- Portable laptop systems vary in beamforming and post-processing.
- Console platforms typically provide the most consistent artifact representation.

#### Machine settings

Standard lung ultrasound settings include:

- Disabling harmonics.
- Minimizing artifact suppression tools.
- Using moderate dynamic range.
- Adjusting depth to include the pleural line and distal artifact zone only.
- Using one focal zone set at or just below the pleural line.
- Proper optimization helps standardize B-line appearance across transducers.

Current veterinary evidence does not support a single optimal frequency setting for lung ultrasound; rather, diagnostically consistent B-line detection has been demonstrated across the native frequency ranges of commonly used transducers, provided adequate penetration and pleural visualization are achieved. In a prospective study using linear (6–14 MHz), microconvex (~5–8 MHz), and phased-array (2–5 MHz) transducers, B-line detection and quantification remained highly consistent across frequency ranges appropriate to each transducer type (4). Accordingly, frequency should be selected within the standard operating range of the chosen transducer and adjusted to ensure visualization of the pleural line and uninterrupted vertical artifact propagation, rather than fixed to a specific numeric value.

### Step 5: apply evidence-based expectations of transducer performance

Based on comparative human and veterinary studies, including recent controlled assessments of vertical artifact morphology (5, 13, 37, 58), clinicians can expect the following performance characteristics:

#### Linear transducer

- Highest pleural-line resolution.
- Best suited for subtle pleural irregularities and small subpleural lesions.
- Penetration limitations in larger dogs.

#### Microconvex transducer

- Balanced depth, resolution, and maneuverability.
- High intercostal flexibility.
- Consistent B-line generation across body sizes.
- Often functions as the primary transducer for VLUS.

#### Curvilinear transducer

- Excellent penetration for medium and large dogs.
- Wide field of view for diffuse disease.
- Slightly less near-field precision than linear or microconvex.

#### Phased-array transducer

- Efficient cardiopulmonary integration.
- Adequate depth and easy intercostal access.
- Less optimal pleural-line detail.

### Step 6: maintain consistency for serial monitoring

When monitoring disease progression or therapeutic response, transducer and frequency consistency is essential for reliable longitudinal comparisons. Changes in transducer geometry or frequency alter vertical artifact shape and can mimic clinical improvement or deterioration. Human ARDS and heart failure monitoring recommendations also emphasize consistent transducer use. Therefore, the baseline transducer should be used for all follow-up scans whenever feasible.

### Step 7: identify when multiple transducers should be used

A comprehensive examination may require more than one transducer:

- Microconvex or curvilinear for global AIS assessment.
- Linear for targeted pleural detail.

This combined approach integrates broad pattern recognition with fine-resolution assessment of the pleural and subpleural regions.

### Step 8: synthesized clinical pathway

A practical bedside decision pathway can be summarized as follows:

- Define the clinical goal.
- Evaluate patient size, chest depth, and BCS.
- Select an initial transducer based on global depth requirements.
- Match transducer to anatomical windows.
- Optimize machine settings to preserve artifact visibility.
- Perform the examination with appropriate expectations for artifact appearance.
- Add a second transducer when pleural detail is needed.
- Maintain transducer consistency when monitoring disease longitudinally.
- This structured framework aligns evidence, anatomy, ergonomics, and technology to support reproducible VLUS practice.

Integration of standardized scanning sites, appropriate patient positioning, and consistent B-line assessment further enhances the reliability of lung ultrasound as a bedside diagnostic tool in small animal emergency and critical care practice.

## Limitations and challenges

Several practical and methodological factors limit consistency, reproducibility, and standardization of VLUS. These challenges span variability among ultrasound systems, transducer-specific technical constraints, operator experience, and the absence of species-specific acquisition guidelines.

### Variability across ultrasound system types

In the current veterinary clinical landscape, clinicians rely on three general categories of ultrasound systems: handheld devices, portable laptop-style units, and larger console platforms. Each introduces distinct limitations that affect LUS reproducibility.

#### Handheld systems

Handheld devices vary widely in imaging technology, including semiconductor “chip” transducers in some platforms and traditional piezoelectric elements in others. These differences affect image resolution, artifact morphology, thermal stability, and overall performance (51). Known limitations include short battery life, device overheating in some non-chip units, reliance on mobile phones or tablets for display, small image size, and transducer footprints that may restrict intercostal or subxiphoid access (44, 63, 69–71). Preliminary veterinary data comparing handheld devices show measurable differences in artifact clarity and image quality even when the same transducer type is used (51).

#### Portable laptop-style units

Portable systems offer improved processing capability and image size, but performance varies across manufacturers. Differences in beamforming, dynamic range, and artifact-suppression algorithms can alter the visibility of B-lines and pleural details, occasionally mimicking transducer-based differences (44, 63).

#### Console-based platforms

Full console systems typically offer the most consistent artifact morphology, the most stable image processing, and the highest overall image quality. Their cost, size, and limited mobility, however, restrict widespread use in many general practice and emergency settings. The resulting variability in equipment across institutions introduces differences in artifact appearance and pleural-line detail that can complicate multicenter comparisons and, in some cases, exceed the variability attributable to transducer selection alone.

### Transducer-specific technical constraints

Although microconvex, curvilinear, linear, and phased-array transducers all reliably detect B-lines, differences in footprint, frequency, and beam geometry influence near-field resolution and the visualization of subtle AIS features. Human studies show that depth, gain, frequency, and focal position alter vertical artifact morphology (72), and veterinary work demonstrates similar effects. Only a limited number of veterinary studies have directly compared different transducer types within the same patient populations, and most investigations primarily evaluate B-line detection rather than broader clinical endpoints such as diagnostic accuracy, therapeutic monitoring, or outcome prediction. Across comparative studies evaluating artifact behavior and pleural-line characterization in dogs and cats, several consistent findings emerge (4, 14, 19, 27, 28, 39):

- Linear transducers provide the highest pleural-line resolution and best depiction of subtle irregularities, narrow vertical artifacts, and small subpleural defects, although their penetration is often insufficient in larger or deep-chested dogs.
- Microconvex transducers balance depth and near-field detail and consistently generate diagnostically valid B-lines but may miss the smallest subpleural irregularities that are visible using higher-frequency linear transducers.
- Curvilinear transducers improve penetration and field of view in large or overweight dogs but have reduced pleural-line sharpness compared with linear or microconvex transducers.
- Phased-array transducers detect AIS-associated vertical artifacts reliably yet provide limited near-field pleural detail because of their narrow sector geometry.
- Switching transducers between serial examinations leads to predictable differences in artifact thickness, brightness, and distal confluence, which can resemble clinical progression or resolution if transducer consistency is not maintained.

These findings show that while AIS detection is transducer-agnostic, the choice of transducer affects pleural-line visualization, early lesion detection, and B-line morphology. Thus, consistency in transducer use should be considered most essential for reliable longitudinal monitoring.

### Operator-dependent variability

Training in veterinary POCUS varies widely across institutions. Some programs provide structured curricula incorporating lung, heart, and abdominal POCUS, whereas others offer minimal exposure. Variability in hands-on experience and case volume contributes to inconsistent proficiency and challenges interobserver agreement. Learning-curve evidence confirms that repeated supervised scanning is required to achieve reliable detection of B-lines and pleural abnormalities (50). Differences in terminology, including variations in the use of “consolidation,” “tissue-like pattern,” and “subpleural lesion,” further reduce communication consistency across centers.

### Lack of standardized protocols and species-specific guidelines

Currently, VLUS lacks uniform recommendations for scanning regions, transducer frequency selection, patient positioning, and the interpretation of “wet” versus “dry” AIS patterns. Variation in thoracic conformation, body condition, and species-specific anatomy also complicates comparisons across studies. Conditions such as pulmonary fibrosis, atelectasis, neoplasia, and chronic inflammatory disease can produce vertical artifacts or pleural irregularities that resemble fluid-associated patterns (13). Without standardized terminology and unified acquisition protocols, distinguishing among etiologies and comparing findings across studies remains challenging.

## Future directions

Future development in AIS detection will depend on improving transducer selection frameworks, refining acquisition protocols, and integrating emerging technologies to enhance diagnostic consistency. Human medicine has well established lung ultrasound protocols and artifact definitions, but comparable veterinary systems remain limited. Species-specific guidelines for dogs and cats that incorporate thoracic shape, body size, and lung depth are needed to reduce variability between clinicians and across institutions, including differences between private practice and academic centers. Broader adoption of handheld and portable ultrasound units will also influence the trajectory of veterinary POCUS by increasing access to bedside imaging and enabling serial lung assessments in emergency, ICU, and field environments (64).

Advances in artificial intelligence and quantitative ultrasound are expected to reduce operator dependency by automating the detection and quantification of B-lines, pleural irregularities, and subpleural consolidations (73–75). Most currently available artificial intelligence models for lung ultrasound analysis have been developed using human imaging datasets. Early veterinary investigations evaluating automated B-line quantification in dogs show encouraging agreement with experienced operators but remain limited in scale. Development of robust veterinary AI systems will require large, expertly annotated datasets from dogs and cats and standardized acquisition protocols. These tools may eventually support decision-assistance systems that guide transducer selection, optimize machine settings, and standardize interpretation. Finally, multicenter veterinary studies, modeled after human LUS research networks, are needed to validate transducer performance, define diagnostic thresholds, and establish unified AIS guidelines that can be broadly implemented across clinical and academic settings.

## Conclusion

Transducer selection directly influences the clarity and consistency of lung ultrasound findings in dogs and cats, particularly for detecting alveolar–interstitial syndrome. Across available evidence, linear, microconvex, curvilinear, and phased-array transducers all identify B-lines and pleural abnormalities, but they differ in near-field resolution, penetration, and field of view. High-frequency linear transducers provide the most precise pleural-line visualization and are valuable for detecting early subpleural pathology. Microconvex and curvilinear transducers offer a balanced combination of depth and maneuverability and remain the most practical choice for comprehensive small-animal examinations. Phased-array transducers support integrated cardiopulmonary assessment but provide less detail at the pleural interface.

Applying an evidence-guided pairing of transducer characteristics with clinical objectives improves interpretive confidence and reduces diagnostic variability. Global AIS assessment is well suited to microconvex or curvilinear transducers, while targeted pleural evaluation benefits from a high-frequency linear transducer when depth permits. Consistent transducer use during serial evaluations minimizes changes in artifact morphology that might otherwise be misinterpreted as clinical progression.

Greater standardization of transducer selection within veterinary curricula and clinical protocols will help reduce interobserver variability and strengthen reproducibility across practices. Further work, including multicenter veterinary studies and integration of quantitative and AI-assisted tools, will refine transducer-specific expectations and support more uniform interpretation. Continued development of species-appropriate, evidence-based guidance is likely to enhance diagnostic accuracy and solidify lung ultrasound as a central modality in small-animal emergency and critical care.
